# The Association between Comorbidities and Comorbid Injuries on Treatment Outcome in Pediatric and Elderly Patients with Injuries in Korea: An Observational Study

**DOI:** 10.3390/ijerph19106277

**Published:** 2022-05-21

**Authors:** Kyunghee Lee, Jieun Hwang

**Affiliations:** 1 Department of Healthcare Management, Eulji University of Korea, 553 Sanseongdaero, Sujeong-gu, Seongnam 13135, Kyeonggi-do, Korea; rose1294@hanmail.net; 2 College of Health Science, Dankook University, 119 Dandaero, Dongnam-gu, Cheonan 31116, Chungcheongnam-do, Korea

**Keywords:** injury, children, elderly, single injury, multiple injuries, Korea National Hospital Discharge Survey

## Abstract

We aimed to compare the characteristics and types of injuries affecting pediatric and elderly patients and to identify factors associated with treatment outcomes. We used data from the 2006–2017 Korea National Hospital Discharge Survey. The patients were divided into two groups, children (0–12 years) and elderly (≥65 years), based on their age at discharge. In total, 47,528 (11,842 children and 35,686 older adults) patients with injuries were identified. The number of deaths and the LOS were 36 (0.3%) and 7.6 days (±10.1), respectively, in the children group, and 861 (2.4%) and 18.5 days (±27.3), respectively, in the elderly group (*p* < 0.001). In the children group, there were increased odds for surgery among boys, Medicaid and health insurance subscribers, patients with multiple injuries, patients without a subdiagnosis, and an increasing number of hospital beds. In the elderly group, there were increased odds for surgery among women, Medicaid and health insurance subscribers, patients who died, patients with a single injury, patients with a subdiagnosis, and increasing numbers of hospital beds. Treatment outcomes could be improved by providing early diagnosis and prompt treatment in pediatric patients and by taking multilateral approaches for multiple injuries and comorbidities in elderly patients.

## 1. Introduction

Advances in medicine have reduced disease-related mortality; however, there has been a consistent increase in injury-related morbidity and mortality [1]. Injury involves three factors, namely, host, agent, and vector and environment, and can be predicted and prevented at three time points: pre-event, event, and post-event [2]. Furthermore, injury cases can be classified according to age (pediatric, adult, and elderly injury); accordingly, there is a need for age-specific prevention policies tailored to specific types and characteristics of injuries. 

Pediatric injury results in substantial individual, social, and national losses and burdens; further, it impairs the quality of life by causing permanent disability [3]. In South Korea, most pediatric injuries are unintentional, including traffic accidents, drowning, and murder [1], with the resulting deaths accounting for more than half of all deaths and mainly occurring at home and in residential areas (38.7%) [4]. The Korean government has established recent measures to raise awareness and prevent traffic accidents, the major cause of injury-related deaths among children, which has resulted in a decrease in traffic accidents involving children [5]. Moreover, measures have been taken to prevent home injury-related deaths among infants and toddlers aged <4 years by changing home environments as well as providing educational and legal interventions for their caregivers [4]. Due to the prevention policies tailored for different types and characteristics of pediatric injury-related deaths, the rate of pediatric injury mortality in Korea has dropped below the Organization for Economic Co-operation and Development average to 2.93. Nevertheless, there has been a continuous increase in the rate of pediatric injuries [6]. In North America, Europe, Australia, and New Zealand, the annual incidence of brain injuries among children and adolescents aged ≤20 years is 691 per 100,000; however, the mortality rate is only 9 per 100,000, with the disability prevalence being higher instead [7]. 

Unlike pediatric injuries, even minor injuries in the elderly progress to severe conditions due to difficulties in recovery, resulting in a tremendous socioeconomic burden given the increased treatment cost [8], cost of support, and sequelae [9]. Injuries in older adults result in worsened visual acuity, hearing, and muscle strength, increasing the risk of falling and slipping. Accordingly, there has been an increase in the rate of hospitalization due to falls among older adults aged ≥65 years, which was 2336 per 100,000 as of 2013 in Korea [9]. In the U.S., the utilization rate of the emergency department (ED) among older adults aged ≥65 years is 458.6 per 10,000; further, the hospitalization and mortality rates were 130 per 10,000 and 5.4 per 10,000, respectively, which indicates that a considerable number of older adults die from falling [10,11]. As aforementioned, it is difficult for older adults to recover from injuries, with falls occurring frequently [12]. Further, injury-related diminished physical functioning adversely affects cognitive function and could cause psychological depression [13]. 

A common characteristic of injuries in children and older adults is that they primarily occur at home since these groups are vulnerable to external risk factors and require care from caregivers [12,14,15]. A study on developing countries, such as Iran, reported that 49.2% of household injuries in children aged <5 years are mild and often result from falling; contrastingly, approximately 50% of these cases are severe injuries that cause serious disabilities [16]. Moreover, falls are the major cause of traumatic brain injury in children aged <4 years. Although they are often non-fatal, unintentional injuries that mostly occur at home, they incur substantial treatment-related social costs [17]. In the U.S., household injuries are the second-most common injuries affecting children aged ≤19 years [18]. Additionally, accidents resulting from falling and slipping at home are common among older adults since they spend most of their time at home and are often alone [19,20,21,22].

Post-injury disabilities in children and older adults affect the work of their caregivers, incur substantial costs due to prolonged treatment, and impair their health-related quality of life [23,24]. Further, these injuries are associated with tremendous social and economic loss as well as a high rate of sequelae and fatality. Accordingly, there has been extensive research on this topic; however, most of these studies were exclusively conducted on specific age groups, including children aged 0–5 years or 0–12 years [25,26,27] and older adults aged ≥65 years [28,29,30]. Moreover, even studies on both pediatric and elderly injuries generally focused on a specific injury [31,32]; injury mortality; or injuries from a specific cause, including traffic accidents [33,34,35,36,37,38,39].

Given the aging population, there is a need for social and national management policies for injuries among older adults and children in dual-income families. Accordingly, there have been studies on the current management and prevention policies for specific injury types according to age (children and older adults). However, there remains unclear evidence regarding between-group differences in the types and characteristics of injuries and treatment outcomes. Obtaining conclusive evidence could inform policymakers in devising tailored prevention and management strategies for these age groups. Therefore, we aimed to analyze the incidence, types, sites, and treatment outcomes of injuries in children and older adults using data from the Korea National Hospital Discharge Survey.

## 2. Materials and Methods

We used data from the 2006–2017 Korea National Hospital Discharge Survey, which is an annual survey conducted by the Korea Disease Control and Prevention Agency (KDCA). This survey seeks to identify the number of discharged patients and their characteristics in order to provide data for informing effective national health policies. The collected data contain information regarding sociodemographic, admission, and disease and treatment characteristics as well as the external cause of injury [40]. After obtaining approval from the Institutional Review Board (IRB) of Eulji University, we obtained permission to extract and use these data for analysis. 

Based on the age at the time of discharge, patients were divided into the children group (0–12 years) or the elderly group (≥65 years). We used the Korean Standard Classification of Disease 7th Version, which is based on the International Classification of Disease 10th Version. Patients with injury were defined as those with a primary diagnosis code of S00–S09 (head injury), S10–S19 (neck injury), S20–S29 (thorax injury), S30–S39 (injury of the abdomen, lower back, and pelvis), S40–S49 (injury of the shoulder and arms), S50–S59 (injury of the elbow and lower arm), S60–S69 (injury of the wrist and hands), S70–S79 (injury of the buttocks and thighs), S80–S89 (injury of the knees and lower legs), S90–S99 (injury of the ankle and feet), T00–T07 (injuries involving multiple body regions), T08–T14 (injuries to an unspecified part of the trunk and limbs), T15–T19 (effects of a foreign body entering through a natural orifice), T20–T32 (burns and corrosions), T33–T35 (frostbite), T36–T50 (poisoning by, adverse effect of, and underdosing of drugs, medicaments, and biological substances), T51–T65 (toxic effects of substances chiefly nonmedicinal as to source), T66–T78 (other and unspecified effects of external causes), or T79 (certain early complications of trauma). In the Korea National Hospital Discharge Survey, each patient is assigned 0–20 subdiagnosis codes. Further, to examine comorbidities in patients with an injury, we classified the subdiagnosis codes based on the disease codes as follows: A00–B99 (certain infectious and parasitic diseases), C00–D48 (neoplasm), D50–D89 (diseases of the blood and blood-forming organs and certain disorders involving the immune mechanism), E00–E90 (endocrine, nutritional and metabolic diseases), F00–F99 (mental and behavioral disorders), G00–G99 (diseases of the nervous system), H00–H59 (diseases of the eye and adnexa), I00–I99 (diseases of the ear and mastoid process), J00–J00 (diseases of the circulatory system), K00–K93 (diseases of the digestive system), L00–L99 (diseases of the skin and subcutaneous tissue), M00–M99 (diseases of the musculoskeletal system and connective tissue), N00–N99 (diseases of the genitourinary system), P00–P96 (certain conditions originating in the perinatal period), Q00–Q99 (congenital malformations, deformations, and chromosomal abnormalities), R00–R99 (symptoms, signs, and abnormal clinical and laboratory findings), NEC T80–T88 (complications of surgical and medical care, NEC), T90–T98 (sequelae of injures, poisoning, and other consequences of external causes), and U00–U19 (provisional assignment of new diseases of uncertain etiology or emergency use). Additionally, patients with and without an injury code as a subdiagnosis were considered to have a single injury and multiple injuries, respectively. 

The chi-square test and *t*-tests were used for between-group comparisons of the number of discharged patients, sex, admission route, insurance type, treatment outcomes, number of hospital beds, and length of stay (LOS) by year. Moreover, we used the chi-square test for between-group comparisons of the incidence, type, place, and treatment outcomes of injuries. Finally, we performed logistic and linear regression analyses to determine the effects of sex, admission route, insurance type, treatment outcomes, number of hospital beds, multiple injuries, and subdiagnosis on surgery and LOS. The results of logistic regression analysis are presented as the adjusted odds ratio results considering all explanatory variables. All statistical analyses were performed using SAS 9.4 software (SAS Institute, Cary, NC, USA). Statistical significance was set at *p* < 0.05. 

## 3. Results

### 3.1. General Characteristics of Patients with Injury

In the study period, there were 11,842 children and 35,686 older adults in the children and elderly group, respectively (Table 1). The children group was predominantly male (7627 [64.4%] boys and 4215 [35.6%] girls), while the elderly group was predominantly female (21,579 [60.5%] women and 14,107 [39.5%] men) (*p* < 0.001). The most common admission route in the children group was the ED (*n* = 7484; 63.2%), followed by outpatient clinics (*n* = 4342, 36.7%) and others (*n* = 16, 0.1%). The most common admission route in the elderly group was the ED (*n* = 23,543, 66.0%), followed by outpatient clinics (*n* = 12,119, 34.0%) and others (*n* = 24, 0.1%). Hospitalization through the ED was more common in the elderly group than in the children group (*p* < 0.001).

In the children group, the most common insurance type was national health insurance (NHI) (*n* = 8863, 74.8%), followed by others (*n* = 2595, 21.9%) and Medicaid (*n* = 384, 3.2%). In the elderly group, the most common insurance type was NHI (*n* = 25,153, 70.5%), followed by others (*n* = 7089, 19.9%) and Medicaid (*n* = 3444, 9.7%). Compared with the children group, the elderly group had a lower proportion of NHI and other insurance types as well as a higher proportion of Medicaid (*p* < 0.001). Regarding treatment outcomes, improvements were observed in most patients in the children (*n* = 11,543, 97.5%) and elderly groups (*n* = 33,076, 92.7%), with only a minor proportion of patients not improving (144 [1.2%] vs. 996 [2.8%]), receiving only a diagnosis (112 [0.9%] vs. 688 [1.9%]), expiring (36 [0.3%] vs. 861 [2.4%]), or others (7 [0.1%] vs. 65 [0.2%]). Compared with the children group, the elderly group showed higher rates of death and non-improvement (*p* < 0.001). The most common number of hospital beds was 500–999 and 100–299 beds in the children (*n* = 4986) and elderly (*n* = 16,774) groups (*p* < 0.001). In the children group, 6313 (53.3%) and 5529 (46.7%) patients lacked and had surgery, respectively, while the corresponding proportions in the elderly group were 19,271 (54.0%) and 16,415 (46.0%) patients, with no significant between-group difference (*p* = 0.191). There was a significant between-group difference in the LOS (7.6 days and 18.5 days in the children and elderly groups, respectively *p* < 0.001). Regarding the annual trends of injuries, the injury incidence declined over the years in the children group, while it either increased or decreased without marked changes in the elderly group (*p* < 0.001).

### 3.2. Characteristics of Injuries

Table 2 shows the injury characteristics. In the children group, the most common injury characteristic was unintentional injury (*n* = 11,711, 98.9%), followed by violence (*n* = 89, 0.8%), unidentified/under investigation (*n* = 29, 0.2%), intentional self-harm (*n* = 9, 0.1%), and unknown (*n* = 4, 0.0%). In the elderly group, the most common injury characteristic was unintentional injury (*n* = 34,410, 96.4%), followed by intentional self-harm (*n* = 800, 2.2%), violence (*n* = 279, 0.8%), unidentified/under investigation (*n* = 163, 0.5%), and unknown (*n* = 34, 0.1%) (*p* < 0.001). In the children group, injuries most commonly occurred in the place of living, followed by road/highway, unknown, school, and sports facility or stadium. Contrastingly, in the elderly group, injuries most commonly occurred in the place of living, followed by road/highway, unknown, farm, and healthcare facility (*p* < 0.001). The most common activity at the time of injury was activities of daily living and unknown in the children and elderly groups, respectively (*p* < 0.001). In the children group, the most common injury mechanism was a fall/slip, followed by traffic accident and bumping, while most cases in the elderly group were caused by a fall/slip (*p* < 0.001).

### 3.3. The Main Diagnoses of Patients with Injury

In the children group, the most common main diagnosis code was for injuries to the head, followed by injuries to the shoulder and upper arm, burn and corrosion, injuries to the knee and lower leg, and injuries to the elbow and lower arm (Table 3). Contrastingly, in the elderly group, the most common main diagnosis code was for injuries to the hip and thigh, followed by injuries to the head; injuries to the abdomen, lower back, and pelvis; injuries to the thorax; and injuries to the knee and lower leg. There were significant between-group differences in the main diagnosis codes (F = 8955.9, *p* < 0.001).

### 3.4. Subdiagnosis of Patients with Injury

Compared with the children group, the elderly group had a higher number of patients with a subdiagnosis (1446 [12.2%] vs. 17,859 [50.0%]) (Table 4). The most common subdiagnosis code in the children group was respiratory disease, followed by certain infectious and parasitic diseases and diseases of the digestive system. Contrastingly, the most common subdiagnosis code in the elderly group was diseases of the circulatory system, followed by diseases of the musculoskeletal system and connective tissue and endocrine, nutritional, and metabolic diseases. The percentage of multiple injuries (inclusion of injury codes in the subdiagnosis) was 36.7% and 39.0% in the children and elderly groups, respectively (*p* < 0.001).

### 3.5. Factors Associated with Surgery in Patients with Injury

In the children group, the factors associated with surgery were sex, insurance type, number of hospital beds, injury codes in subdiagnosis, and subdiagnosis codes (Table 5). Specifically, the odds for surgery were 1.194 times higher among boys than among girls (95% confidence interval (CI) 1.101–1.295), 2.719 times higher among patients with NHI than among those with other insurance (95% CI 2.447–3.022), and 2.719 times higher among patients with Medicaid than among patients with other insurance (95% CI 2.164–3.417). Compared with children hospitalized in a hospital with 100–299 beds, those hospitalized in hospitals with 500–999 beds and ≥1000 beds had 1.74 times (95% CI 1.584–1.912) and 2.074 times (95% CI 1.797–2.394) higher odds for surgery. Furthermore, patients with a single injury and without a subdiagnosis had decreased odds for surgery than those with multiple injuries and with a subdiagnosis, respectively (adjusted odds (aOR) 0.514, 95% CI 0.472–0.559 and aOR 0.578, 95% CI 0.511–0.653, respectively). 

In the elderly group, the factors associated with surgery were sex, insurance type, treatment outcomes, number of hospital beds, injury codes in subdiagnosis, and subdiagnosis codes. Specifically, the odds for surgery were lower among men than among women (aOR 0.796, 95% CI 0.76–0.834). Further, the odds for surgery were 1.573 and 1.993 times higher among patients with Medicaid (95% CI 1.437–1.722) and NHI (95% CI 1.873–2.121) compared with those with other insurance types. Compared with patients with “other” treatment outcomes, the odds for surgery were 5.125 and 2.883 times higher among patients with improvement (95% CI 2.761–9.514) and those who died (95% CI 1.529–5.437), respectively. Additionally, the odds for surgery were positively correlated with the hospital size. The odds for surgery were 0.584 times lower among patients with multiple injuries than among those with a single injury (95% CI 0.557–0.613); further, they were 1.164 times higher among patients with a subdiagnosis than among those without (95% CI 1.113–1.217). 

### 3.6. Factors Associated with LOS in Patients with Injury

In the children group, the factors associated with LOS were insurance type, number of hospital beds, injury codes in subdiagnosis, subdiagnosis codes, and surgery (Table 6). Specifically, the LOS was shorter among patients with NHI or Medicaid compared with that of patients with other insurance types (e.g., automobile insurance, worker’s compensation); moreover, it was negatively correlated with the number of hospital beds. The LOS was higher among patients with multiple injuries, subdiagnoses, and surgery compared with among patients with a single injury, without a subdiagnosis, and without surgery, respectively. 

In the elderly group, the factors associated with LOS were sex, insurance type, number of hospital beds, injury codes in subdiagnosis, subdiagnosis codes, and surgery. Specifically, the LOS was shorter among men than among women as well as among patients with NHI or Medicaid than among patients with other insurance types (e.g., automobile insurance and worker’s compensation). The LOS was negatively correlated with the number of hospital beds. Further, the LOS was higher among patients with multiple injuries, subdiagnoses, and surgery compared with among patients with a single injury, without a subdiagnosis, and without surgery, respectively. 

## 4. Discussion

This study compared the types and characteristics of injuries in children and older adults in Korea as well as the factors associated with treatment outcomes in the past 12 years. In both groups, there were high rates of admission through the ED, NHI subscribers, improved outcomes, and patients who did not undergo surgery. Contrastingly, the children group had more boys and more hospitalizations in 500–999-bed hospitals, while the elderly group had more women and more hospitalizations in 100–299-bed hospitals. 

The finding of a high proportion of women in the elderly group is consistent with other reports in the U.S., Canada, and Taiwan [41]. This could be attributed to the longer life expectancy for women than for men, which results in a greater proportion of female patients in the elderly group. Specifically, since 2002, female patients are consistently discharged more often than male patients in Korea [42]. However, we observed higher odds for surgery among older men than among older women, which suggests that men are more vulnerable to injuries. Since falls that cause hip and thigh injuries frequently occur among older adults, the outcomes of falls may differ according to physical sexual dimorphism, including height and body weight. This indicates that men are at a greater risk of developing sequelae and disabilities after an injury than women. Taken together, there is a need for sex-specific preemptive interventions at time of admission to improve treatment outcomes. 

In both groups, the most common places of injuries were place of living and roads/streets. However, there were between-group differences in the other common places of injuries; specifically, children frequently incurred injuries in play and cultural facilities, while older adults were frequently injured in healthcare facilities. Generally, most pediatric injuries were unintentional injuries, including those caused by traffic accidents, drowning, burns, and falls. These injuries may result in acquired disabilities or death, which could cause tremendous social and economic loss [43]. Accordingly, various national and international measures have been implemented to improve safety in common places of pediatric injuries. 

Prevention of pediatric injuries requires accurate assessment of the current status, scientific evidence-based research, safety education and campaigns, and enactment and enforcement of regulations for creating a safe environment [44]. Specifically, regulations such as mandatory seatbelts, helmets, and fences are considered powerful measures for preventing injury. In fact, enforcing mandatory helmet use when riding a bicycle increased the rate of helmet usage [45], which reduced the occurrence of head injuries [46]. 

In Korea, safety signs, speed bumps, and speed safety cameras are installed in child safety zones to ensure slow driving and prevent traffic accidents [47]. Specifically, “Minshik’s law” was enacted in 2019, which stipulates that drivers who cause traffic accidents in child safety zones receive aggravated punishment [48]. Additionally, the act on safety management of child play facilities was enacted to allow children to safely and comfortably use the playground [49], and therefore prevent accidents. Further studies are warranted to determine whether these systems have positive influences on the incidence of accidents and the resulting pediatric injuries and deaths in order to identify effective safety measures. 

The head and hip/thigh were the most common injury sites in the children and elderly groups, respectively, which are closely associated with the types of activities that cause injury [50]. Children often injure themselves during leisure activities, i.e., playing, and from falling or bumping into something during their normal day-to-day lives. Contrastingly, older adults often injure themselves during activities of daily living due to impaired gait and balance or from falling in healthcare and long-term care facilities [50,51]. Pediatric injuries are heavily influenced by environmental factors [52]; therefore, ameliorating the environment can prevent these injuries. In fact, the injury rate differs according to the targeted direction of policies [53]. Regarding the annual trends of injuries, both groups showed a marked reduction in the incidence of injury in 2013, which could be attributed to the development and dissemination by the KDCA in 2013 of evidence-based safety guidelines for preventing injuries [9].

Injuries among older adults in healthcare facilities can also be prevented, and therefore preventive measures are required [51]. Falls can be caused by intrinsic factors related to the deterioration of physical and mental functions as well as by extrinsic factors involving the physical and situational environments [54]. Falls are common among older adults and frequently occur in healthcare facilities. In fact, it is possible to predict and prevent a considerable percentage of falls. Accordingly, healthcare facilities have established various preventive strategies, including prevention activities targeting inpatients, multidisciplinary team efforts, and the use of fall prevention tools [55]; however, the rate of falls remains high. Falls frequently occur within 1–5 days of admission and contribute to extending the LOS [56]; therefore, it is important to thoroughly educate patients and caregivers upon admission. Moreover, there is a need for hospital-wide efforts to improve patient safety, including staff training and environmental management. 

Even a minor injury can cause permanent disability among children, which impairs the quality of their remaining lives [57]. Moreover, injury-induced mobility problems in older adults deteriorate their quality of life and cause them to require care at other healthcare or nursing facilities, which results in additional socioeconomic costs. In fact, medical cost is strongly associated with injury severity [58]; furthermore, the burden of medical cost influences patients even after discharge. The increasing number of elderly patients resulting from the aging population has led to an increased incidence of injuries. Moreover, there is an increase in the number of older adults (age ≥ 80 years) with injury [59], which is suggestive of an increased medical cost burden among older adults with an injury. Our findings demonstrated a between-group difference in the LOS by approximately four fold. As suggested by the between-group difference in the number of hospital beds, older adults with injury are generally treated through long-term hospitalization in smaller hospitals and clinics. This indicates that older adults may require a relatively long time to recover from even minor symptoms or that they may require continuous management due to permanent sequelae or disability after injury treatment. In Taiwan, the mean LOS of elderly patients (age ≥ 65 years) with injury was 9.1 days (±12.0) [60], which is markedly shorter than that in Korea. Since extended hospital stay by patients with injuries aggravates caregiver burden and strains the health insurance finances, there is a need for tailored measures targeting specific age groups and hospital sizes. 

Subdiagnosis, which was identified as a factor associated with LOS, was more common in the elderly group than in the children group. Respiratory and infectious diseases (acute diseases that occur during treatment) were more common comorbidities in the children group. Contrastingly, chronic diseases (e.g., heart disease and endocrine disease) and musculoskeletal disorders were more common in the elderly group. Chronic conditions increase the risk for complications, which increases the disease severity; therefore, it is important to prevent complications by managing chronic diseases [61]. Furthermore, given the annual increase in the population of older adults [62], there is a need for tailored prevention measures and post-care management measures for older adults. 

Prevention policies and education specific to different injury types (e.g., injury site and mechanism) in children and older adults can facilitate injury prevention. Specifically, given the increasing incidence of severe injuries among older adults, who often have multiple chronic conditions, it is important to establish post-care management strategies for preventing injury and minimizing socioeconomic loss. Moreover, although both groups require care in their daily lives, they often receive inadequate care due to the increase in dual-income households and the older adult population. Accordingly, there is a need for public caregiving policies tailored to various injury characteristics [63].

Since we analyzed only patients hospitalized due to an injury, we excluded patients who received medical care in outpatient clinics or the ED. Nonetheless, our findings may inform the establishment of tailored injury prevention policies. Another strength of this study is that our findings demonstrate the significance of managing comorbidities, which were factors associated with the mean LOS and surgery. 

However, this study has several limitations. First, patients with an injury were defined only based on their main diagnosis. Therefore, we excluded patients with an injury code only in their subdiagnosis, which could have led to the underestimation of the number of patients with an injury. Nonetheless, we additionally categorized patients into those with a single injury or multiple injuries based on whether their subdiagnosis codes included a code for injury. Second, we examined only a small number of factors associated with surgery and LOS due to the limitations of the study data. Third, we did not review the risk of coding errors in the recording of the diagnosis codes, which is a limitation of analyzing extensive datasets. Fourth, this study is an analysis of secondary data, which did not include information on whether the main diagnosis and sub-diagnosis existed at the time of admission. In fact, hospitals in South Korea often do not check or record the present on admission (POA). Even if it is recorded, there are differences in the methods for collecting information between hospitals, raising concerns in terms of the stability of the collected information and the reliability of the information. For this reason, this study assumed that the disease that the patient had at the time of admission and the disease that occurred after admission would equally affect patients’ death and length of hospitalization. It is necessary that follow-up studies resolve the limitations of the data in the current study by preparing a method to distinguish whether a disease was present at the time of admission and by examining the difference in results.

## 5. Conclusions

We aimed to compare the characteristics and types of injuries affecting pediatric and elderly patients and to identify factors associated with treatment outcomes. In the children group, there were increased odds for surgery among boys, Medicaid and health insurance subscribers, patients with multiple injuries, and patients without a subdiagnosis, and with an increasing number of hospital beds. In the elderly group, there were increased odds for surgery among women, Medicaid and health insurance subscribers, patients who died, patients with a single injury, and patients with a subdiagnosis, and with an increasing number of hospital beds. According to these results, treatment outcomes could be improved by providing early diagnosis and prompt treatment in pediatric patients and by taking multilateral approaches for multiple injuries and comorbidities in elderly patients.

## Figures and Tables

**Table 1:** Sociodemographic characteristics of discharged pediatric and elderly patients.

	0–12 Years (*n* = 11,842)		≥65 Years (*n* = 35,686)		*p*-Value ^†^
	*n*	%	*n*	%	
Sex					<0.001
Male	7627	64.4	14,107	39.5	
Female	4215	35.6	21,579	60.5	
Admission route					<0.001
Emergency department	7484	63.2	23,543	66.0	
Outpatient department	4342	36.7	12,119	34.0	
Others	16	0.1	24	0.1	
Insurance type					<0.001
NHI	8863	74.8	25,153	70.5	
Medicaid	384	3.2	3444	9.7	
Others	2595	21.9	7089	19.9	
Treatment outcome					<0.001
Improved	11,543	97.5	33,076	92.7	
Not improved	144	1.2	996	2.8	
Diagnosis only	112	0.9	688	1.9	
Others	7	0.1	65	0.2	
Death	36	0.3	861	2.4	
Number of hospital beds					<0.001
100–299	3819	32.2	16,774	47.0	
300–499	1846	15.6	4779	13.4	
500–999	4986	42.1	12,039	33.7	
≥1000	1191	10.1	2094	5.9	
Surgery					0.191
No	6313	53.3	19,271	54.0	
Yes	5529	46.7	16,415	46.0	
Length of stay					<0.001 ^‡^
Mean ± standard deviation	7.6	10.1	18.5	27.3	
Year of injury (children/elderly) *					<0.001
2006 (0.02%/0.08%)	1661	14.0	3400	9.5	
2007 (0.02%/0.07%)	1456	12.3	3143	8.8	
2008 (0.01%/0.06%)	1199	10.1	3149	8.8	
2009 (0.01%/0.06%)	1061	9.0	2999	8.4	
2010 (0.01%/0.06%)	1181	10.0	3298	9.2	
2011 (0.01%/0.05%)	1036	8.7	3015	8.4	
2012 (0.01%/0.05%)	993	8.4	3071	8.6	
2013 (0.01%/0.04%)	737	6.2	2584	7.2	
2014 (0.01%/0.04%)	638	5.4	2500	7.0	
2015 (0.01%/0.04%)	569	4.8	2385	6.7	
2016 (0.01%/0.03%)	507	4.3	2341	6.6	
2017 (0.01%/0.05%)	804	6.8	3801	10.7	

^†^ chi-square test, ^‡^ *t*-test, * It is the ratio of the number of patients to the population.

**Table 2:** Injury characteristics in the children and elderly groups.

	0–12 Years (*n* = 11,842)		≥65 Years (*n* = 35,686)		*p*-Value ^†^
	*n*	%	*n*	%	
Intentionality					<0.001
Unintentional	11,711	98.9	34,410	96.4	
Intentional self-harm	9	0.1	800	2.2	
Violence	89	0.8	279	0.8	
Unidentified/under investigation	29	0.2	163	0.5	
Unknown	4	0.0	34	0.1	
Place of injury					<0.001
Home	3406	28.8	10,397	29.1	
Road/highway	3073	26	10,289	28.8	
Unknown	2810	23.7	10,038	28.1	
Farm	16	0.1	1177	3.3	
Water, ocean, outdoors	183	1.5	825	2.3	
Healthcare facility	25	0.2	512	1.4	
Industrial, construction sites	13	0.1	484	1.4	
Commercial areas	255	2.2	471	1.3	
Group residential facility	16	0.1	464	1.3	
Other	186	1.6	354	1	
Play, cultural, and public Facilities	408	3.4	231	0.6	
Other traffic areas	32	0.3	207	0.6	
School	852	7.2	124	0.3	
Sports facilities and stadiums	567	4.8	113	0.3	
Activity during injury occurrence					<0.001
Unknown	2757	23.3	12,437	34.9	
In daily life	2773	23.4	8829	24.7	
While moving	1460	12.3	5403	15.1	
During other specified activities	1393	11.8	4271	12	
During paid work	2	0	2107	5.9	
During unpaid work	3	0	1051	2.9	
During a leisure activity	2413	20.4	741	2.1	
During treatment	20	0.2	412	1.2	
While drinking alcohol	41	0.3	280	0.8	
During a sports game	431	3.6	102	0.3	
During education	549	4.6	53	0.1	
Mechanism of injury					<0.001
Falling, slipping	4102	34.6	19,812	55.5	
Traffic accident	3178	26.8	9005	25.2	
Bumping	1704	14.4	1771	5	
Poisoning	244	2.1	1456	4.1	
Other	485	4.1	1448	4.1	
Unknown	161	1.4	1020	2.9	
Stabbing, cutting, severed	417	3.5	557	1.6	
Fire, flame, heat	1452	12.3	532	1.5	
Asphyxiation	42	0.4	70	0.2	
Drowning	46	0.4	14	0	
Gun shot	10	0.1	1	0	
Sexual violence	1	0	0	0	

^†^ chi-square test.

**Table 3:** Main diagnosis codes in children and elderly patients with injury.

		Main Diagnosis			
		0–12 years (*n* = 11,842)		≥65 Years (*n* = 35,686)	
		Cases	%	Cases	%
S70–S79	Injuries to the hip and thigh	325	2.7	6401	17.9
S00–S09	Injuries to the head	3324	28.1	6280	17.6
S30–S39	Injuries to the abdomen, lower back, and pelvis	456	3.9	5305	14.9
S20–S29	Injuries to the thorax	106	0.9	5175	14.5
S80–S89	Injuries to the knee and lower leg	1049	8.9	2727	7.6
S50–S59	Injuries to the elbow and lower arm	958	8.1	2172	6.1
S40–S49	Injuries to the shoulder and upper arm	1576	13.3	1895	5.3
S10–S19	Injuries to the neck	272	2.3	1180	3.3
S60–S69	Injuries to the wrist and hands	860	7.3	1093	3.1
T51–T65	Toxic effects of substances chiefly nonmedicinal as to source	120	1	985	2.8
S90–S99	Injuries to the ankle and foot	667	5.6	853	2.4
T20–T32	Burns and corrosions	1555	13.1	594	1.7
T36–T50	Poisoning by, adverse effect of, and underdosing of drugs, medicaments, and biological substances	118	1	471	1.3
T00–T07	Injuries involving multiple body regions	57	0.5	173	0.5
T15–T19	Effects of foreign body entering through natural orifice	272	2.3	158	0.4
T66–T78	Other and unspecified effects of external causes	89	0.8	99	0.3
T08–T14	Injuries to the unspecified part of trunk and limbs	29	0.2	93	0.3
T79	Certain early complications of trauma	8	0.1	29	0.1
T33–T35	Frostbite	1	0	3	0

**Table 4:** Subdiagnosis codes in children and elderly patients with injury.

Subdiagnosis		0–12 Years (*n* = 11,842)		≥65 Years (*n* = 35,686)		*p*-Value
		Cases	%	Cases	%	
Subdiagnosis						<0.001
	No	10,396	87.8	17,827	50.0	
	Yes	1446	12.2	17,859	50.0	
Disease codes						
	A00–B99	165	1.4	1267	3.6	<0.001
	C00–D48	10	0.1	762	2.1	
	D50–D89	69	0.6	940	2.6	
	E00–E90	24	0.2	5349	15.0	
	F00–F99	60	0.5	2173	6.1	
	G00–G99	112	0.9	1660	4.7	
	H00–H59	78	0.7	614	1.7	
	H60–H95	57	0.5	314	0.9	
	I00–I99	37	0.3	10,508	29.4	
	J00–J99	744	6.3	3181	8.9	
	K00–K93	151	1.3	3248	9.1	
	L00–L99	97	0.8	834	2.3	
	M00–M99	69	0.6	6042	16.9	
	N00–N99	27	0.2	2415	6.8	
	P00–P96	14	0.1	0	0.0	
	Q00–Q99	34	0.3	24	0.1	
	R00–R99	137	1.2	1218	3.4	
	T80–T88	47	0.4	282	0.8	
	T90–T98	4	0.0	100	0.3	
	U00–U19	7	0.1	80	0.2	
Injury code						<0.001
	Single	7491	63.3	21,762	61.0	
	Multiple	4351	36.7	13,924	39.0	

**Table 5:** Factors associated with surgery in children and elderly patients with injury.

Variables	0–12 Years (*n* = 11,842)			≥65 Years (*n* = 35,686)		
	aOR	95% CI		aOR	95% CI	
Sex						
Men	1.194	1.101	1.295	0.796	0.76	0.834
Women	Ref.			Ref.		
Admission route						
Emergency department	0.391	0.122	1.252	0.916	0.393	2.136
Outpatient department	0.625	0.195	1.999	0.877	0.376	2.046
Others	Ref.			Ref.		
Insurance type						
NHI	2.719	2.447	3.022	1.993	1.873	2.121
Medicaid	2.719	2.164	3.417	1.573	1.437	1.722
Others	Ref.			Ref.		
Treatment outcome						
Improved	2.221	0.37	13.321	5.125	2.761	9.514
Not improved	0.521	0.081	3.335	0.927	0.486	1.767
Diagnosis only	0.139	0.019	1.004	0.39	0.197	0.769
Death	3.15	0.46	21.573	2.883	1.529	5.437
Others	Ref.			Ref.		
Number of hospital beds						
≥1000	2.074	1.797	2.394	2.659	2.407	2.938
500–999	1.74	1.584	1.912	1.989	1.89	2.093
300–499	0.982	0.869	1.109	1.395	1.303	1.492
100–299	Ref.			Ref.		
Injury code						
Multiple	0.514	0.472	0.559	0.584	0.557	0.613
Single	Ref.			Ref.		
Subdiagnosis						
Yes	0.578	0.511	0.653	1.164	1.113	1.217
No	Ref.			Ref.		

aOR, adjusted odds; CI, confidence intervals.

**Table 6:** Factors associated with length of stay in the children and elderly patients with injury.

Variables	0–12 Years (*n* = 11,842)			≥65 Years (*n* = 35,686)		
	β	t	*p*-Value	β	t	*p*-Value
Intercept	5.227532	1.23	0.2184	14.03701	2.26	0.0239
Sex						
Men	−0.19788	−1.1	0.271	−1.08321	−3.77	0.0002
Women	Ref.			Ref.		
Admission route						
Emergency department	3.437655	1.47	0.1424	0.220338	0.04	0.9668
Outpatient department	1.598193	0.68	0.4954	−4.65999	−0.88	0.3788
Others	Ref.			Ref.		
Insurance type						
NHI	−4.83614	−21.69	<0.0001	−9.4027	−24.99	<0.0001
Medicaid	−2.94505	−5.71	<0.0001	−5.52255	−9.88	<0.0001
Others	Ref.			Ref.		
Treatment outcome						
Improved	0.272504	0.08	0.9386	2.962697	0.92	0.3575
Not improved	−3.89227	−1.07	0.2825	−2.90815	−0.88	0.381
Diagnosis only	−3.62249	−0.99	0.3203	−3.25628	−0.97	0.333
Death	−5.77275	−1.49	0.1353	2.061551	0.62	0.5362
Others	Ref.			Ref.		
Number of hospital beds						
≥1000	−1.78143	−5.59	<0.0001	−3.78238	−6.18	<0.0001
500–999	−1.29049	−6.12	<0.0001	−1.6089	−4.97	<0.0001
300–499	0.063464	0.24	0.8128	1.410254	3.3	0.001
100–299	Ref.			Ref.		
Injury code						
Multiple	3.889958	20.38	<0.0001	5.039694	16.54	<0.0001
Single	Ref.			Ref.		
Subdiagnosis						
Yes	5.837476	22.01	<0.0001	8.649975	31.03	<0.0001
No	Ref.			Ref.		
Surgery						
Yes	3.952947	21.36	<0.0001	11.11277	38.24	<0.0001
No	Ref.			Ref.		
*R* ^2^	0.150434			0.097606		
F-value	139.6			257.21		
*p*-value	<0.0001			<0.0001		

## Data Availability

The datasets analyzed during the current study are available at the following website: https://www.kdca.go.kr/contents.es?mid=a20303010502 (accessed on 8 March 2021).
